# Study protocol of Branch Atheromatous Disease-related stroke (BAD-study): a multicenter prospective cohort study

**DOI:** 10.1186/s12883-022-02976-9

**Published:** 2022-12-09

**Authors:** Shengde Li, Jun Ni, Xiaoyuan Fan, Ming Yao, Feng Feng, Dongxue Li, Jianxun Qu, Yicheng Zhu, Lixin Zhou, Bin Peng

**Affiliations:** 1 Department of Neurology, Peking Union Medical College Hospital, Peking Union Medical College and Chinese Academy of Medical Sciences, Beijing, China; 2 State Key Laboratory of Complex Severe and Rare Diseases, Peking Union Medical College Hospital, Chinese Academy of Medical Science and Peking Union Medical College, Beijing, China; 3 Department of Radiology, Peking Union Medical College Hospital, Peking Union Medical College and Chinese Academy of Medical Sciences, Beijing, China; 4 Research Scientist, Siemens Healthineers, Beijing, China

**Keywords:** Branch atheromatous disease, Stroke, Prospective cohort, Risk factors, Early neurological deterioration, High-resolution magnetic resonance imaging, Prognosis

## Abstract

**Background:**

As a meaningful subtype of ischemic stroke in Asians, Branch atheromatous disease (BAD)-related stroke is associated with high early neurological deterioration (END) and disability, but is understudied and without recommended therapy. The mechanism of END still remains unclear. Branch atheromatous disease-related stroke study (BAD-study) therefore aims to investigate demographic, clinical and radiological features, and prognosis of BAD-related stroke in Chinese patients.

**Methods/design:**

BAD-study is a nationwide, multicenter, consecutive, prospective, observational cohort study enrolling patients aged 18–80 years with BAD-related stroke within 72 h after symptom onset. Initial clinical data, laboratory tests, and imaging data are collected via structured case report form, and follow-ups will be performed at 7 days, 30 days, 90 days, 6 months and 12 months after enrollment. The primary outcome is the score on modified Rankin Scale at 90-day follow-up with single-blinded assessment. Secondary outcomes include END within 7 days, and National institute of health stroke scale score, Barthel index, cerebrovascular events, major bleeding complications, and all-cause mortality during 90-day follow-up. Characteristics of penetrating and parent artery will be assessed by high-resolution magnetic resonance imaging combined with other imaging techniques.

**Discussion:**

BAD-study can provide demographic, clinical, radiological, and prognostic characteristics of BAD-related stroke, and thereby potentially figure out the vascular mechanism of early neurological deterioration and optimize therapy strategy with the aid of advanced imaging technique. Baseline data and evidence will also be generated for randomized controlled trials on BAD-related stroke in the future.

## Background

Stroke leads to millions of death and disability in China and other countries annually [1, 2]. To optimize stroke management and improve functional outcome, causative mechanism of stroke is of great importance. Branch Atheromatous Disease (BAD), first described by Caplan in 1989 [3], is increasingly becoming a clinical entity with the aid of neuroimage, characterized by stenosis or occlusion at the origin of a penetrating artery resulting from atherosclerosis and leading to ischemic lesion [4]. In our study, the term “Branch Atheromatous Disease (BAD)-related stroke” is used for ischemic stroke due to branch atheromatous disease.

Although proposed as a meaningful concept, BAD remained neglected and underused in clinical practice, researches and guidelines for the past three decades [4]. Epidemiological data on BAD is spare, and most studies were reported in Asian population [4]. Small vessel occlusion or intracranial atherosclerosis are more common in Asians, which is different from Western populations. BAD-related stroke accounted for 9.1% in Japan and 20.4% in Hongkong among ischemic stroke patients [5, 6]. Perforating arteries involve lenticulostriate artery (LSA), paramedian pontine artery (PPA), thalamoperforating artery, anterior choroidal artery and Heubner's artery [4, 6], while what have been widely studied Is LSA and PPA, indirectly via features of ischemic lesions [4, 7]. Based on clinical and neuroimage evidence, neurologists and researchers have proposed the diagnostic criteria for BAD-related stroke [7–9].

Of patients with BAD-related stroke, the mean age ranged from 54 to 75 years, slightly younger than that in large artery disease (LAD) or lacunar infarction (LACI), and male gender was more prevalent [4, 6, 10]. The median National Institutes of Health Stroke Scale (NIHSS) score of BAD-related stroke was 4, lower than 6 in LAD, but similar with that in LACI. The short-term disability (modified ranking scale [mRS] ≥ 3) rates of LAD, BAD-related stroke, and LACI were 74%, 61%, and 47%, respectively [6]. However, long-term outcomes were comparable between BAD-related stroke and LACI [11]. Finally, the clinical, neuroradiologic, and predictive data are insufficient for BAD-related stroke.

One challenge of BAD-related stroke was its high incidence of early neurological deterioration (END) [5, 12], which was strongly associated with poor outcome [13]. Compared with distal single subcortical infarction, BAD-related stroke showed higher-volume lesion (median, 1.79 vs 0.44 ml) and more frequent END [10, 14–16]. The rate of END following intravenous thrombolysis was estimated as 13.8%, measured by the deterioration of NIHSS score at 24 h (≥ 4) [12]. Intravenous thrombolysis seemed unable to prevent END and controversial about its effect on improving functional outcome among patients with BAD-related stroke [12, 17–19]. In addition, Seners P, et al. found that strongest predictors for END were consistent across thrombolysed and non-thrombolysed acute ischemic stroke patients including hyperglycemia, proximal arterial occlusion, large infarcts, no recanalization or re-occlusion, and etc. [12]. Thus, preventing the progression of stenosis and re-occlusion of proximal penetrating artery has become the essential node of the early management of BAD-related stroke. Multiple studies focused on the mechanism of END due to unique brain ischemia with poor collateral circulation [20]. However, no evidence-based therapy was recommended for secondary preventive treatment in acute phase of BAD-related stroke.

Previous study found that dual-antiplatelet or anticoagulation therapy might reduce END, but their effect on BAD-related stroke remained unclear and might increase the risk of bleeding [21, 22]. Tirofiban also showed efficacy on reducing END and improving outcomes in patients with endovascular therapy, intravenous thrombolysis or BAD-related stroke, but warranting further confirmation in large-sample patients with BAD-related stroke [23–25]. The optimal regimen for reducing END and improving outcome remains inconclusive [19].

In addition, many researchers focused on the role of inflammatory indicators in stroke pathogenesis and prognosis, including the effect of white blood cell, platelet, lipid level and etc. [12, 26–28]. For instance, a randomized controlled study reported that high-dose statins could reduce the level of inflammatory indicators and improve the outcome of ischemic stroke [29]. Elevated high-sensitivity C-reactive protein and inflammatory indicators also predicted poor outcome of stroke [30, 31]. However, the effect of inflammatory indicators on BAD-related stroke was unknown.

Another advance in BAD-related stroke is the application of high-resolution magnetic resonance imaging (HR-MRI) and images of vessel wall, which facilitates visualized analysis of LSA, PPA and their parent arteries [32, 33], and the further research of vascular pathophysiology of BAD-related stroke and END.

Although neurologists increasingly consider BAD-related stroke as a clinical entity and a subtype of ischemic stroke with relatively poor outcome, its demographic, clinical and radiological characteristics are still lacking with little evidence of therapy. Therefore, we describe here the protocol of BAD-study, a multicenter prospective cohort study in China to investigate demographic, clinical, radiological features, risk factors, and prognosis of BAD-related stroke and explore the optimal regiment for good outcome.

## Methods/design

### Study design

BAD-study is a nationwide, multicenter, consecutive, prospective, observational, cohort study recruiting patients with BAD-related stroke within 72 h after symptom onset. Due to February 28, 2022, this ongoing study has recruited 22 hospitals with the equipment of 3 Tesla (T) magnetic resonance imaging, with the leader of Peking Union Medical College Hospital in Beijing, China. BAD-study was approved by the Ethics Committee of Peking Union Medical College Hospital on May 25, 2021 (No. ZS-2982B), and written informed consents (model consent form) are required for all patients. In addition, protocol modifications, if any, would be approved by the ethics committee. The anticipated duration of the study will be about 2 years from June 1, 2021 until May 31, 2023. BAD-study has been registered on July 28, 2021 at clinicaltrials.gov, with identifier NCT04973774.

### Study objectives

The primary objective of this study is to figure out the demographic, clinical, radiological characteristics, risk factors, and prognosis of BAD-related stroke in this clinical-radiological cohort with large sample, and evaluate the effects of different real-word therapies on outcome. Secondary objectives are to: 1) investigate the vascular mechanism of END; 2) establish the association between clinical, clinical, radiological characteristics and clinical outcome and model for predicting outcome; 3) find out the standardized process and criteria for analyzing penetrating arteries using vessel wall imaging (VWI) based on 3 Tesla-MRI; 4) explore the efficacy of safety of specific drugs, such as aspirin and tirofiban, providing baseline data for further clinical trials.

### Study population

All consecutive ischemic stroke patients aged 18–80 years visiting any center of our study considering BAD-related stroke will be screened for eligibility according to the following inclusion and exclusion criteria. All eligible patients will be Invited to participate in this observational study.

- Inclusion criteria:
- 1. Age:18–80 years.
- 2. Acute cerebral infarction, if the clinical manifestations are transient, new infarct lesion should be found on Diffusion Weighted Imaging (DWI) at the same time.
- 3. The time from symptom onset to enrollment is less than 72 h. If the onset time was unknown, the time of last known free of new ischemic symptoms to enrollment is less than 72 h.
- 4 Meet all the following radiological criterial:
  - 1) DWI lesion: single (isolated) deep (subcortical) infarct;
  - 2) The culprit vessels are the LSA or PPA, and the infarct lesion on DWI conforms to one of the following characteristics (A/B):
  - A. LSA: (1) “Comma-like” infarct lesions with “Fan-shaped” extension from bottom to top in the coronary position; OR (2) ≥ 3 layers (layer thickness 5–7 mm) on axial DWI images of the head;
  - B. PPA: the infarct lesion extends from the deep pons to the ventral pons on the axial DWI of the head.
- 3) No ≥ 50% stenosis on the parent artery of the criminal vessel (i.e. corresponding basilar or middle cerebral artery) (confirmed by magnetic resonance angiography [MRA] or computed tomography angiography [CTA] or digital substraction angiography [DSA]).
- 5 Signed informed consent by the patient or legally authorized representatives.
- Exclusion criteria:
- 1. Intracranial hemorrhagic diseases, vascular malformations, aneurysms, brain abscesses, malignant space occupying lesions or other non-ischemic intracranial lesions observed by baseline head CT and MRI, MRA/CTA/DSA.
- 2. There was ≥ 50% stenosis of extracranial vessels with ipsilateral serial relationship.
- 3. Cardiogenic embolism: atrial fibrillation, myocardial infarction, valvular heart disease, dilated cardiomyopathy, infective endocarditis, atrioventricular block disease, heart rate less than 50 beats /min.
- 4. Have received or plan to receive acute endovascular treatment after onset of the disease.
- 5. Stroke caused by other clear causes, such as moyamoya disease, arterial dissection, vasculitis, etc.
- 6. mRS score prior to the onset of the disease was ≥ 2 points.
- 7. Known malignant tumor.
- 8. Life expectancy ≤ 6 months.
- 9. Contraindications of 3 T MRI examination.
- 10. Pregnant or lactating women.
- 11. Participation in another clinical within 3 months before enrollment, or taking part in another ongoing study.

### Procedures

**Fig. 1:**
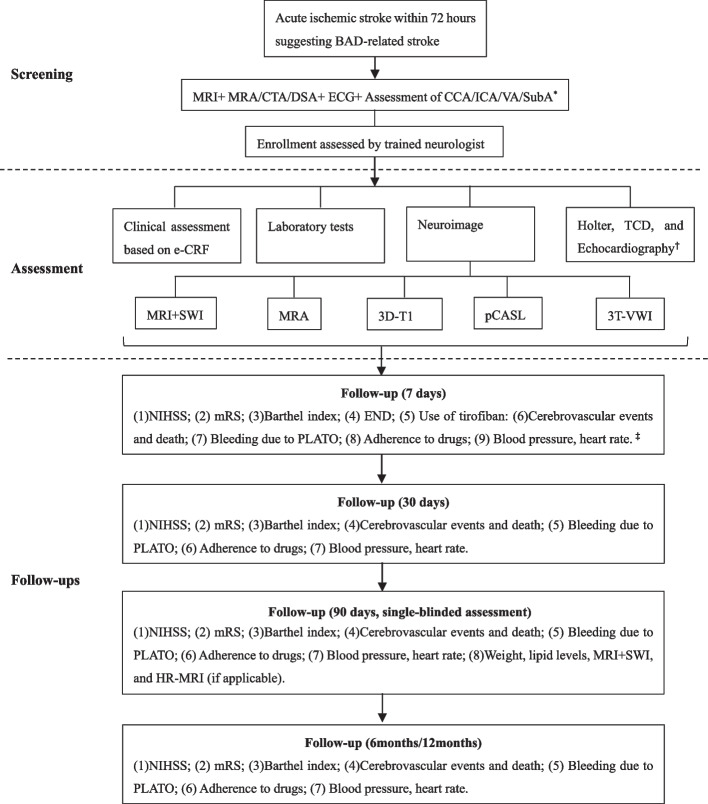
The flowchart of BAD-study. BAD: Branch atheromatous disease; CTA: Computed tomography angiography; DSA: Digital subtraction angiography; e-CRF: Electronic case report form; END: Early neurological deterioration; HR-MRI: high-resolution magnetic resonance imaging; MRA: Magnetic resonance angiography; MRI: Magnetic resonance imaging; mRS: Modified Ranking scale; NIHSS: National institute of health stroke scale; pCASL: pseudo-continuous arterial spin labelling; PLATO: Platelet Inhibition and Patient Outcomes; SWI: Susceptibility weighted imaging; VWI: Vessel wall imaging. ^*^ CCA: Common carotid artery; ECG: Electrocardiograph; ICA: Internal carotid artery; VA: Vertebral artery; SubA: Subclavian artery; ^†^ TCD: Transcranial doppler; ^‡^ Drugs include Antiplatelet drugs, lipid-lowering drugs including statins, anticoagulant drugs, antihypertensive drugs, antidiabetics, and other neuroprotective drugs

The board of primary investigator, neurologists, radiologists, epidemiologists, pharmacologist and statisticians was established on June 1, 2021. All hospital personnel have been trained on the reading of neuroimages in screening stage, the collection of data at baseline and each follow ups, and etc. The enrollment of first case in each center will be confirmed by the board. Eligible patients will be recruited at the time of signing the informed content. Baseline data included demographic and clinical assessments, neuroimages and laboratory data (first ones after stroke onset). Follow-ups will be conducted at 7 days, 30 days, 90 days, 6 months, and 12 months after enrollment, via face-to-face interviews or telephone, when the mRS, cerebrovascular events, adverse effects of medications, and etc. will be evaluated. At the follow-up of 7 days, the event of END will be assessed since stroke onset. The 90-day follow-up is required to be assessed by the neurologists, who are blinded to the clinical, radiological characteristics and treatment of selected patients. The flowchart of BAD-study is shown in Fig. 1.

### Study measurements

#### Clinical data

Data of patients will be collected and reported using the electronic Case Report Form (e-CRF) at baseline and follow-up periods. The quality of data will be monitored dynamically by the central center. Clinical items include demographics, medical history, history of medication, clinical presentations, and treatments in hospital. The blood pressure and intake and output volume within 72 h after enrollment are also recorded. The details is shown in Table 1.

**Table 1:** Clinical assessment of BAD-related stroke

Items	Assessment
Demographics	Age, gender
Medications prior to stroke	Antiplatelet drugs, statins, anticoagulant drugs, and tirofiban
Medical history	Stroke, hypertension, diabetes mellitus, dyslipidemia, heart disease, chronic respiratory disease, autoimmune diseases, blood system disorders, and thyroid disorder
Personal history	Smoking, alcohol use, and daily activity
Family history	Cardiovascular and cerebrovascular diseases
Current stroke onset	
Time information	Time of symptom onset, arrival at hospital, and enrollment
Stroke symptom	Types (ischemic stroke or transient ischemic attack [TIA]), and presentation (pure motor hemiparesis, pure sensory, sensorimotor syndrome, and others)
Blood pressure	(1) Arrival at hospital, enrollment, and each follow up; (2) Monitor of blood pressure within 72 h after enrollment (≥ 3 times per day, interval time ≥ 2 h)
Heart rate	Arrival at hospital, enrollment, and each follow up
Intake and output volume	Monitor of intake and output volume within 72 h after enrollment
Stroke severity	National Institutes of Health Stroke Scale
Functional assessment	mRS
Activities of daily living	Barthel index
Reperfusion therapy	Data of intravenous thrombolysis, and change of NIHSS score
Medications after stroke	Antiplatelet drugs, lipid-lowering drugs including statins, anticoagulant drugs, antihypertensive drugs, antidiabetics, and other neuroprotective drugs
END	Therapies for END, and time of END occurrence
Use of tirofiban	Time, dosage, duration and side effects

*END* Early neurological deterioration, *TIA* Transient ischemic attack, *mRS* Modified Ranking scale, *NIHSS* National institute of health stroke scale

#### Laboratory tests

According to Chinese guidelines and clinical practice [34], initial clinical biochemistry routine tests including total blood cell count, liver and renal functions, lipid levels, and blood coagulation test will be recorded under standard lab procedures. If applicable, autoimmune biomarkers, homocysteine concentrations, glycosylated hemoglobin, fasting glucose, thyroid gland function, high sensitivity C reactive protein, Protein C, Protein S, Antithrombin III, and activated protein C will be measured at baseline. If applicable, lipid levels will be measured at 90-day follow up.

### Imaging protocols

#### MRI

The MRI studies are conducted on 3 T MR scanners (GE Discovery 750 or SIEMENS Vida). Three dimensional (3D) T1, MRA, high-resolution vessel wall imaging and pseudo-continuous arterial spin labelling (ASL) are conducted, and the parameters are shown in Table 2. Based on Table 2, the imaging parameters are modified and verified at each center, if applicable. In addition, routine T1-weighted, T2-weighted, fluid-attenuated inversion recovery (FLAIR), diffusion-weighted imaging, apparent diffusion coefficient (ADC) and susceptibility weighted imaging (SWI)/T2*WI will also be performed, using the standardized protocol in each center.

**Table 2:** MRI parameters of vascular imaging

Scanner	Sequences	TR/TI/TE (ms)	FOV (mm)	Matrix	FA	Number of slices	Slice thickness (mm)	LD/PLD (ms)	Time
SIEMENS	T1-SPACE	900/-/14	170 × 170	288 × 288	-	240	0.6	-	8 min 56 s
	3D-T1 (MPRAGE)	2300/900/3.5	256 × 232	256 × 232	8	192	1	-	4 min 40 s
	TOF-MRA	20.7/-/3.8	216 × 174	432 × 324	20	216	0.4	-	6 min 20 s
	ASL	4550/-/12	210 × 210	60 × 60	180	36	4	1800/2000	2 min 7 s
GE	T1-CUBE	800/-/15.8	204 × 184	320 × 256	-	248	0.8	-	4 min 45 s
	3D-T1 (Bravo)	6.7/400/2.9	256 × 230	256 × 256	12	170	1.0	-	4 min 11 s
	TOF-MRA	16/-/2.1	220 × 165	352 × 224	20	176	1.2	-	3 min 28 s
	ASL	4886/-/10.5	240 × 240	64 × 64		40	4	1450/2025	4 min 44 s

*ASL* Arterial spin labeling, *FA* Flip angle, *FOV* Field of view, *LD* Labeling duration, *PLD* Post-labeling delay, *TE* Echo time, *TI* Inversion time, *TOF-MRA* Time-of-flight MR angiography, *TR* repetition time

#### Other image techniques

Other assessment techniques for cerebral artery, carotid artery, vertebral artery, and subclavian artery, including MRA, CTA, DSA or vascular ultrasound are conducted according the standardized protocol in each center. In addition, the results of Holter, transcranial doppler sonography, and ultrasonic cardiogram will be recorded based on the criteria at each center, if applicable.

#### Image analysis and classification

The BAD-related stroke will be divided as two groups: 1) Lesion in area of LSA; and 2) Lesion in area of PPA. The plaque, stenosis, vessel wall, and cerebral perfusion of PPA and LSA will be assessed. The vascular lesion sites of penetrating artery and its parent artery are grouped into three types: 1) Plaque within parent artery blocking the branch orifice; 2) Plaque extending into the branch from parent artery; 3) Plaque originating in orifice of branch [3]. The length, thickness, square, volume, and signal of plaque will be measured. In LSA analysis based on 3 T-VWI, the number, length, and shape of LAS will also be evaluated [35].

The process of image collection, storage, synthesis and analysis are guided by a committee including senior radiologists and neurologists. All images will be evaluated by two experienced neurologists and will be reviewed by senior neurologist J Ni in case of disagreement.

### Outcome measures

The primary outcome in our study is the score on the modified Rankin scale at 90 days, which is a 7-point scale ranging from 0 (no symptoms) to 6 (death) [36]. Secondary outcomes include END within 7 days, and NIHSS score, Barthel index, cerebrovascular events (myocardial infarction, new-onset ischemic stroke, new-onset intracranial hemorrhage), and major bleeding complications measured by PLATO (Platelet Inhibition and Patient Outcomes) definition during 90-day follow-up [37]. All-cause mortality within 90 days is also recorded. The follow up times are extended to 1 year, as the long-term outcome of BAD-related stroke remains inconclusive [4, 6].

The END is defined as: 1) From symptom onset to 7 days after enrollment; and 2) Occurrence of deterioration of neurologic deficits after initial assessment: A. An increase of the NIHSS score ≥ 4 points or the NIHSS motor score ≥ 1 point for ischemic patients; or B. The stereotyped attacks still repeat ≥ 3 times after arrival at hospital or progress to persistence status for patients with internal capsule warning syndrome or pontine warning syndrome [12, 38].

### Sample size

There is no specific hypothesis for the primary outcome in our cohort study, thus, the sample size is estimated based on the number of variables, using the method recommended for registry study (10 times number of variable) [39]. The sample size is estimated as at least 450, and increases to 495 for 10% loss during follow-up.

### Statistical analysis

Categorical variables will be presented as frequencies and percentages, such as gender, vascular risk factors, lesions of penetrating artery, and therapies. Continuous variables with non-normal distributions will be shown as median and interquartile range (IQR), while continuous variables with normal distributions as mean and 95% confidence interval (CI), such as age, NIHSS score, blood pressure, and hemoglobin concentration. For the statistical significance analysis of the outcomes, Pearson χ 2 or Fisher’s exact test, Wilcoxon tests, and t-tests will be used, where appropriate, with statistical uncertainty expressed by means of 95% CI. Cox proportional hazards model, Logistic regression model, and Poisson regression model will be used to assess the association between outcomes and clinical or radiological predictors, where appropriate. The good prognosis is defined as 0–2 mRS score, and poor prognosis as 3–6 mRS score at 90 days. Predictors including clinical and radiological features and different treatments will be evaluated. All analysis will be performed using SAS 9.4 and two-sided *P* < 0.05 is considered significant.

### Data management

As mentioned above, the metadata of BAD-study are stored in the structured eCRF via internet, with central monitoring dynamically. Only anonymous data will be provided to researchers. In addition, data monitoring committee is not needed in this observational study.

### Current status

The first patient of BAD-study was enrolled on August 16, 2021, after thorough assessment by the board. Until March 13, 2022, our ongoing study has recruited 143 patients. The brief demographic and clinical data of eligible patients are shown in Table3.

**Table 3:** Baseline characteristics of patients enrolled in BAD-study

Characteristic	Total
Overall, no.	143
Age, years, mean (95%CI)	59.6 (25.9–93.3)
Female sex, no. (%)	44 (30.8)
Types of stroke onset, no. (%)	
Ischemic Stroke	140 (97.9)
TIA	2 (2.1)
History of stroke, no. (%)	35 (24.5)
Ischemic stroke	33 (23.1)
TIA	1 (0.7)
Cerebral hemorrhage	1 (0.7)
History of hypertension, no. (%)	82 (57.3)
History of diabetes mellitus, no. (%)	44 (30.8)
Elevated LDL-C (Self-report), no. (%)	3 (2.1)
Smoking, no. (%)	
Never	78 (54.5)
Ever	65 (45.5)
Regular drinking, no. (%)	
Never	100 (69.9)
Ever	43 (30.1)
NIHSS score at enrollment, median (IQR)	3 (2–6.3)
Hours from symptom onset to enrollment, median (IQR)	48 (30–65)

*CI* Confidence interval, *IQR* Interquartile range, *TIA* Transient ischemic attack, *NIHSS* National institute of health stroke scale

## Discussion

BAD-study will be the first prospective clinical-radiological cohort with long-term follow-up and large sample, focusing on the BAD-related stroke, a neglected but clinically meaning subtype of stroke in Asians with poor collateral circulation and high disability [4, 10, 40]. The demographic clinical, radiological, and prognostic characteristics of BAD-related stroke in China will be reported.

In addition, we noticed that the BAD-related stroke was categorized as “stroke of undetermined etiology” in TOAST classification system, and thus failed to reflect its underlying stroke mechanism [4, 6, 40]. Our study will update the original TOAST classification, and prove the concept of BAD-related stroke. In addition, we noticed the higher frequency of cardiogenic embolism in elderly patients with lacunar infarcts, and excluded patients with cardiogenic stroke in our study [41].

With the documented data of laboratory tests, the role of inflammatory indicators on the prognosis of stroke will be assessed. The management of inflammatory indicators might be a novel therapy on reducing END and improving the outcome of BAD-related stroke [12, 28, 29, 31].

With the aid of MRI, MRA, HR-MRI, and ASL, BAD-study will analyze the cerebral artery, vessel wall of parent artery and penetrating artery, artery plaques, brain tissue, cerebral perfusion, and volume of lesion in BAD-related stroke. In addition, visualized analysis of LSA, and its association with outcomes will help neurologists understand the vascular mechanism of END [35, 42]. Radiological data of artery and lesion will provide clues for possible effective drugs. Moreover, quantitative analysis will be conducted to analyze the effect of lesion on the volume of gray matter and white matter, as global brain inflammation could be elicited by localized lesion [43, 44]

Besides, the comparison of different therapy strategies on primary outcome and END will provide direct clinical evidence to optimize the acute therapy in BAD-related stroke [13] and find the probably effective strategy of preventing END [12]. Based on the results of BAD-study, a risk prediction model will be generated for the prognosis, which may help neurologists individualize treatment strategy.

BAD-study will also assess the efficacy and safety of tirofiban, providing baseline data for further randomized controlled trials in patients with BAD-related stroke. And the diagnostic criteria of BAD-related stroke will be verified in clinical practice [7–9].

## Data Availability

The data of BAD-study will be available from the corresponding author on reasonable request.

## Abbreviations

ADC: Apparent diffusion coefficient
ASL: Arterial spin labeling
BAD: Branch atheromatous disease
CCA: Common carotid artery
CI: Confidence interval
CTA: Computed tomography angiography
DSA: Digital subtraction angiography
DWI: Diffusion weighted imaging
e-CRF: Electronic case report form
ECG: Electrocardiograph
END: Early neurological deterioration
FA: Flip angle
FLAIR: Fluid attenuated inversion recovery
FOV: Field of view
HR-MRI: High-resolution magnetic resonance imaging
ICA: Internal carotid artery
IQR: Interquartile range
LACI: Lacunar infarction
LAD: Large artery disease
LD: Labeling duration
LSA: Lenticulostriate artery
MRA: Magnetic resonance angiography
MRI: Magnetic resonance imaging
mRS: Modified Ranking scale
NIHSS: National institute of health stroke scale
pCASL: Pseudo-continuous arterial spin labelling
PLATO: Platelet Inhibition and Patient Outcomes
PLD: Post-labeling delay
PPA: Paramedian pontine artery
SWI: Susceptibility weighted imaging
SubA: Subclavian artery
T: Tesla
TCD: Transcranial Doppler
TE: Echo time
TI: Inversion time
TIA: Transient ischemic attack
TOF-MRA: Time-of-flight MR angiography
VA: Vertebral artery
TR: Repetition time
VWI: Vessel wall imaging
